# Spondylodiscitis complicated by paraspinal abscess in a 10-year-old child

**DOI:** 10.1590/0037-8682-0134-2021

**Published:** 2021-04-28

**Authors:** Maria Francesca Gicchino, Nicoletta di Maio, Anna Di Sessa

**Affiliations:** 1 University of the Study of Campania “Luigi Vanvitelli”, Department of Woman, Child and General and Specialized Surgery, Napoli, Italy.

A 10-year-old girl presented to our department with a one-month history of back pain and limp. Initially, inflammatory spondyloarthropathy was diagnosed, and anti-inflammatory treatment was prescribed. Given the absence of improvements, the patient underwent magnetic resonance imaging (MRI) of the spine, which revealed morphostructural alterations in the median and parasagittal areas of both L3-L4 intervertebral disk and L3 and L4 vertebral bodies and edema of the same vertebrae. Pathological tissue in the left paravertebral region and iliopsoas, with descending involvement up to L5, were detected. These findings were compatible with spondylodiscitis with associated phlegmon in the left paravertebral area extending to the iliopsoas (Figure 1). Therefore, spondylodiscitis with paraspinal abscess was diagnosed^1^. A lumbar corset was prescribed, and treatment with broad-spectrum antibiotics based on intravenous clindamycin and ceftriaxone for three weeks, followed by oral cefditoren and clindamycin for five weeks was prescribed^1^. After treatment, the patient’s condition improved. Three months later, a control MRI showed resolution of L3-L4 spondylodiscitis with no vertebral edema and normalization of the previously altered signal and the inflammatory tissue in the left paravertebral area involving the iliopsoas (Figure 2). In the orthopedic evaluation, the patient did not present back pain, and the use of a corset was stopped. 

**FIGURE 1:**
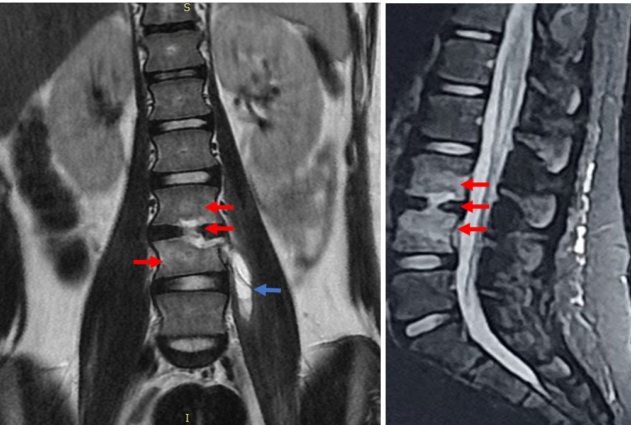
Spondylodiscitis with abscess in left paravertebral region extending to iliopsoas.

**FIGURE 2:**
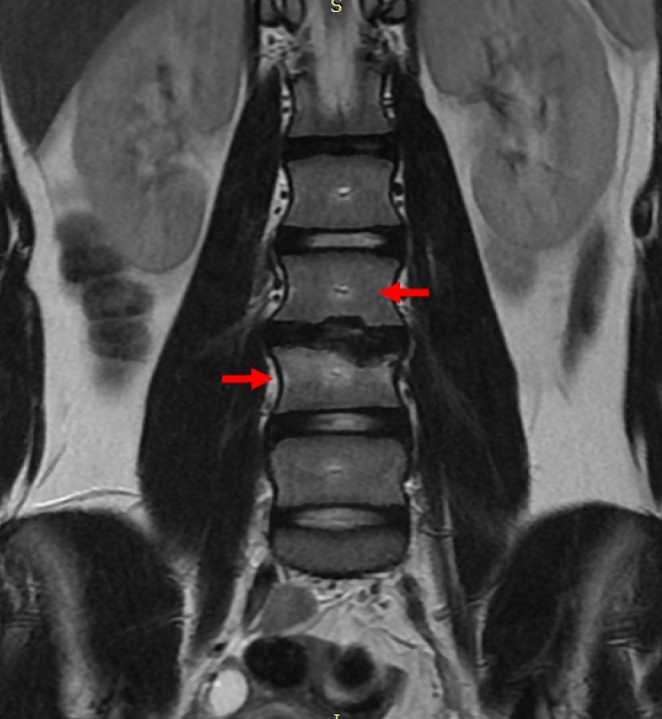
Resolution of spondylodiscitis and paravertebral abscess.

Spondylodiscitis is rare in childhood, and its symptoms are nonspecific. It can be misdiagnosed as bone tumors, fractures, or inflammatory arthropaties^2^. Diagnostic delay can provoke complications. Patients with back pain should be investigated to avoid potential diagnostic delays or misdiagnosis^3^.

## References

[1] Kang HM, Choi EH, Lee HJ, Yun W, Lee CK, Cho TJ (2016). The etiology, clinical presentation and long-term outcome of spondylodiscitis in children. Pediatr Infect Di J.

[2] Mylona E, Samarkos M, Kakalou E, Fanourgiakis P, Skoutelis A (2009). Pyogenic vertebral osteomyelitis: a systematic review of clinical characteristics. Semin Arthritis Rheum.

[3] Principi N, Esposito S (2016). Infectious Discitis and Spondylodiscitis in Children. Int J Mol Sci.

