# Erratum

**DOI:** 10.1590/0037-8682-0134b-2021

**Published:** 2021-06-02

**Revista da Sociedade Brasileira de Medicina Tropical/Journal of the Brazilian Society of Tropical Medicine**

**Title:** Spondylodiscitis complicated by paraspinal abscess in a 10-year-old child 

**54: (e0134-2021) 2021 - Page: 1 - doi:** 10.1590/0037-8682-0134-2021 

Correct indexing of authors:

Maio, Nicoletta di

Sessa, Anna Di

**Should read:**

Di Maio, Nicoletta

Di Sessa, Anna

